# Body mass index–measured adiposity and population attributability of associated factors: a population-based study from Buea, Cameroon

**DOI:** 10.1186/s40608-016-0139-8

**Published:** 2017-01-07

**Authors:** Leopold Ndemnge Aminde, Jeannine A. Atem, Andre Pascal Kengne, Anastase Dzudie, J. Lennert Veerman

**Affiliations:** 1 The University of Queensland, School of Public Health, Herston, QLD 4006 Australia; 2 Non-communicable Diseases Unit, Clinical Research Education, Networking & Consultancy (CRENC), Douala, Cameroon; 3 Faculty of Health Sciences, University of Buea, Buea, Cameroon; 4 South African Medical Research Council and University of Cape Town, Cape Town, South Africa; 5 Department of Medicine, Douala General Hospital and Faculty of Medicine & Biomedical Sciences, University of Yaoundé, Yaoundé, Cameroon

**Keywords:** Overweight, Obesity, Body mass index, Prevalence, Cameroon

## Abstract

**Background:**

Obesity is currently a global health challenge driven by a mix of behavioural, environmental and genetic factors. Up to date population-based disease burden estimates are needed to guide successful prevention and control efforts in African countries. We investigated the prevalence and population attributable fractions of overweight and obesity in Buea, the Southwest region of Cameroon.

**Methods:**

Data are from a community-based cross-sectional study involving randomly selected adults conducted in 2016. Body mass index (BMI) was categorized according to the WHO classification. Multivariable logistic regressions were used to investigate factors independently associated with obesity. Corresponding population attributable fractions were estimated.

**Results:**

Among the 1,139 participants, age-standardized prevalence (95% CI) of overweight and obesity were; 36.5 (33.7–39.3) and 11.1 (9.3–12.9) percent respectively. Mean BMI was 25.3 ± 4.3 kg/m^2^; women were heavier than men (25.8 vs. 24.4 kg/m^2^; *p* <0.0001). Factors associated with obesity were; female gender [odds ratio 3.20 (95% CI: 1.93–5.59)], age > 31 years [3.21 (1.86–5.28)] and being married [2.10 (1.60–3.51)]. At the population level; older age, being married, low level of education, high monthly income and physical inactivity accounted respectively for 11.9%, 21.8%, 11.6%, 6.4% and 8.7% of overweight and obesity among the women, while older age and being married explained 9.2% and 28.3% of overweight and obesity in men.

**Conclusion:**

The prevalence of overweight and obesity in this semi-urban Cameroonian population is high, affecting over a third of individuals. Community-based interventions to control weight would need to take into account gender specificities and socio-economic status.

## Background

Overweight (body mass index [BMI] > 25 kg/m^2^) and obesity (BMI > 30 kg/m^2^) are conditions of excessive fat accumulation that may impair health. According to the World Health Organization (WHO) 2014 estimates, about 2 billion of the world’s adult population are overweight with over 600 million being obese [1]. Overweight and obesity are recognized public health problems especially in industrialized countries [2–4] with prevalence up to almost 40% [5]. The prevalence is now rapidly increasing in low-income settings and Africa in particular, due to rapid urbanization and the adoption of western lifestyles [6, 7]. Current global trends from pooled analysis of population based studies around the world suggest an almost three-fold increase in age-standardized prevalence of obesity (from 3.2–10.8% and 6.4–14.9% for men and women respectively) over the last four decades [8].

In sub-Saharan Africa, South Africa seems to carry a significant burden of obesity [9] with adult population prevalence of over 30%, largely due to its comparatively higher level of urbanization. In Nigeria, Okpechi et al. using the WHO STEPwise approach in a population-based cross-sectional study in Abia State found prevalence of overweight or obesity of 33.7% [10]. Similarly, a population survey among 1,521 Nigerian adults found overweight and obesity rates of 32.2% and 19.6% for men and 29.8% and 36% for women respectively [11]. Data from Ghana among financial institution workers showed over half of participants were either overweight (37.8%) or obese (17.8%), with physical inactivity, being married, female gender and increasing age being independently associated with increased body mass [12]. In Cameroon, Fezeu et al. showed an almost doubling of the age-standardized prevalence of overweight and obesity in rural settings between 1994 and 2003 [13]. More recently, Fouda and colleagues found a prevalence of obesity of 23.4% among workers in Douala [14]. While studies in Cameroon have explored obesity, the majority are from specific sub-populations [14–17] and a few from the general population conducted over a decade ago [13, 18]. The most recent population level estimates for adult overweight and obesity in Cameroon are from a study conducted in 2010 in an urban setting [19].

In SSA, nutrition transition, rapid urbanization and increasing sedentary behaviours have been earmarked as drivers for the obesity epidemic [20, 21]. Cameroon, a lower middle income country like other SSA countries is experiencing similar trends in urbanization (urban growth rate of 3.5%) [22] and westernization of diets. Data from a 2007 nationally representative household consumption and expenditures survey (ECAM3) among women and children in Cameroon suggested consumption of sugar of up to 68.7% (for household) and 78.3% (individually) during the week prior to survey [23]. In addition, data from Food and Agricultural Organization (FAO) suggest that per capita dietary intake increased from 2,001 kilocalories per day (Kcal/Day) in 1962 to 2,269 (Kcal/Day) in 2007, during which an almost double fat consumption was noticed (26–43 g per day). In 2011, dietary intake had further increased to 2586 (Kcal/Day) with sugar and oil intakes of 104 and 132 (Kcal/Day) respectively [24]. Socioeconomic status and physical inactivity are other important risk factors of the obesity epidemic and poor metabolic health previously described in Cameroon [13, 18, 25]. These observations highlight that obesity is rapidly taking a significant position as a cause of disease burden in Cameroon and may worsen if nothing is done. The population attributable risk estimates for these and other potential drivers in Cameroon have not been investigated. Population attributable fraction (PAF) or population attributable risk percent refers to the proportion of cases (disease or outcome) that would not occur in a population if the factor (risk factor or exposure) were eliminated [26]. Its calculation requires the prevalence of exposure to a given risk factor in the population, and the relative risk (RR) of the outcome associated with that exposure. BMI is the most widely used parameter to define overweight and obesity, especially for clinical practice and for epidemiological studies. In this study, we assessed the current adult prevalence of BMI-defined overweight and obesity in Buea, Cameroon and the population attributable impact of its determinants.

## Methods

### Study design and population

During April 2016, we conducted a community based cross-sectional study in Buea, a semi-urban town and Capital of the Southwest region of Cameroon. The study population consisted of adults (age ≥ 18 years) residing in Buea. The Buea Health District (BHD) has seven health areas and an overall population of about 200,000 inhabitants [27]. The estimated sample size was generated using an online sample size calculator [28] and the following criteria: confidence level of 95%, precision of 3%. This gave an estimated sample size of 1,061 subjects.

Via a multi-stage sampling technique, eligible participants were recruited. Systematic random sampling was used to identify households (every fourth house was selected) in each health area to be included in the survey. Thereafter, for each selected household, a simple random sampling via balloting of eligible members was used to select one participant from each house. Data collectors alternated between daytime and evening visits to homes during recruitment to optimize inclusion of individuals who were likely to be at work place during the day.

### Assessment of demographic and health parameters

Data were collected by trained final year medical students to minimise error. This included data on socio-economic and demographic characteristics (age, sex, marital status, level of education, monthly income), and lifestyle and personal health (alcohol use, smoking history, family history of cardiovascular disease, consumption of healthy food (rich in vegetables and fruits, low in salt and saturated fats). Participants who had difficulties with respect to details of food content were further clarified by the data collectors. Physical measurements were done using standard methods. Weight was measured using Seca® scales while height was measured using adult Leicester® stadiometers with participants in light clothing and barefoot. With the stadiometers against the wall, participants stood upright without shoes and their heels and occiput on the stadiometer. Measurement was to one decimal place (for weight in kg) and to the nearest 0.5 cm (for height). Body mass index of participants was calculated via the following formula: *weight (kg) / height (m)* ^*2*^ *.* Participants’ BMI was further categorised according to the WHO International Classification of adult normal, overweight and obesity: 18.5 ≤ BMI (kg/m^2^) ≤ 24.99 as normal weight, 25.0 ≤ BMI (kg/m^2^) ≤ 29.99 as pre-obese, 30.0 ≤ BMI (kg/m^2^) ≤ 34.99 as Obese class I, 35.0 ≤ BMI (kg/m^2^) ≤ 39.99 as Obese class II and BMI ≥ 40.0 kg/m^2^ as obese class III. Overall, participants with BMI ≥ 25.0 kg/m^2^ and BMI ≥ 30.0 kg/m^2^ were considered overweight and obese respectively [29].

### Ethical issues

This study was approved by the Ethical Committee of the Southwest Regional Delegation of the Ministry of Public Health (MOH) of Cameroon. The components and purpose of the study were explained to participants and only those who freely provided consent were included. Confidentiality was maintained and the study adhered to the Helsinki declarations.

### Data analysis

We used IBM-Statistical Package for Social Sciences statistical software v.23 for Windows (SPSS Inc., Chicago, IL) and STATA software version 12 SE for analysis of the data. Via direct standardization, we used the Cameroon National population in 2016 [30] as standard population to calculate the age-standardized prevalence of overweight and obesity. Overall, categorical variables were summarised with counts and percentages while continuous variables were summarized using means and standard deviations. The chi square test and independent samples t-test were used for group comparisons where appropriate. To determine factors associated with overweight and obesity, we used binary logistic regressions. In the first step, bivariate analysis was done using the following predictor variables; age (dichotomized as <32 years and ≥ 32 years, to explore the effect of obesity on either side of the mean population age), gender (male vs. female), monthly income (<50,000FCFA ~ USD100, 50,000–100,000FCFA ~ USD 100–200, and >100,000FCFA ~ USD 200), level of education [low-moderate (up to secondary/high school) vs. high (diploma/university)], marriage (yes or no), smoking (never, former or current smoker), alcohol consumption (yes or no) and physical activity status (≥3 times per week vs. <2 times per week). All variables with 25% significance (i.e. *p* ≤ 0.25) were included in the multivariable model to determine independent associations. We have presented adjusted odds ratios (aOR) and 95% confidence intervals of significant associations in the multivariable analysis. Statistical significance was set at *p* < 0.05.

### Population attributable fraction calculation

To obtain the RR of each of the significant independent factors identified, the above model was similarly constructed as a Poisson regression with robust variance estimator. These adjusted RRs were used in the estimation of the PAF. This was done because the odds ratio obtained from a logistic regression of a relatively common disease or outcome potentially overestimates the RR [31]. Further to this, we used a formula (eq. 1) which provides valid estimates of PAF in the presence of confounding [26, 32]. We estimated the uncertainty (95% confidence intervals) around our PAF estimates based on the Bonferroni inequality with formula (eq. 2) [33].

1 $$ \mathbf{P}\mathbf{A}\mathbf{F} = \mathbf{P}\ \left(\mathbf{R}\mathbf{R}-\mathbf{1}/\mathbf{R}\mathbf{R}\right) $$

where P = prevalence of outcome exposed to given risk factor (exposure) and RR = adjusted relative risk.

2 $$ \left[{\mathbf{P}}_{\mathbf{L}}\left(\mathbf{R}{\mathbf{R}}_{\mathbf{L}}-\mathbf{1}/\mathbf{R}{\mathbf{R}}_{\mathbf{L}}\right),\ {\mathbf{P}}_{\mathbf{U}}\left(\mathbf{R}{\mathbf{R}}_{\mathbf{U}}-\mathbf{1}/\mathbf{R}{\mathbf{R}}_{\mathbf{U}}\right)\right] $$

where P_L_ is the lower limit of the 97.5% CI of P, RR_L_ is the lower limit of the 97.5% CI of RR, P_U_ and RR_U_ the respective upper limits.

## Results

### General characteristics of the study population

Socio-demographic characteristics of the study population are presented in Table 1. Overall, 1,139 of the 1,250 adults invited were analysed in this study (response rate = 91.1%). Overall, the mean age was 32 years with the majority (68%) being young adults (aged 18–34 years). Women accounted for 61% of the study population. A quarter of the participants were married and 46% had less than secondary or high school. Over a tenth of the population were unemployed, affecting mostly women (11.6% vs. 12.3%; *p* < 0.0001). With respect to monthly income, over two-thirds (64%) of the population had a monthly income less than XAF 50,000 (~ USD 100), women being disadvantaged (66.1% vs. 60.7%; *p* < 0.0001).

**Table 1:** Socio demographic characteristics of study population according to gender

Characteristics	Male (*n* = 437, 38.3%)	Female (*n* = 702, 61.7%)	Total (*N* = 1,139)	*p*-value
Age, mean ± SD	31.4 ± 12.8	31.6 ± 12.1	31.5 ± 12.4	0.747
BMI, mean ± SD, kg/m^2^	24.4 ± 3.4	25.8 ± 4.7	25.3 ± 4.3	<0.0001
Age category	*n* = 437	*n* = 702	*N* = 1,139	0.032
18–24 years	188 (43.0)	261 (37.1)	449 (39.4)	
25–34 years	109 (24.9)	216 (30.7)	325 (28.5)	
35–44 years	63 (14.4)	106 (15.1)	169 (14.8)	
45–54 years	56 (12.8)	79 (11.2)	135 (11.8)	
55–64 years	11 (2.5)	35 (5.0)	46 (4.0)	
≥ 65 years	10 (2.3)	5 (1.0)	15 (1.4)	
Marital Status	*n* = 437	*n* = 702	*N* = 1,139	<0.0001
Single	338 (77.3)	427 (60.8)	765 (67.2)	
Divorced	11 (2.5)	29 (4.1)	40 (3.5)	
Widowed	11 (2.5)	33 (4.7)	44 (3.9)	
Married	77 (17.6)	213 (30.3)	290 (25.4)	
Level of education	*n* = 435	*n* = 699	*N* = 1,134	0.002
Up to secondary	89 (20.5)	209 (29.9)	298 (26.3)	
High school	98 (22.5)	135 (19.3)	233 (20.5)	
Diploma	41 (9.4)	85 (12.2)	126 (11.1)	
Undergrad University	171 (39.3)	225 (32.2)	396 (34.9)	
Postgrad University	36 (8.3)	45 (6.4)	83 (7.2)	
Employment status	*n* = 429	*n* = 700	*n* = 1,129	<0.0001
Unemployed	50 (11.6)	84 (12.0)	134 (11.9)	
Retired	18 (4.2)	10 (1.4)	28 (2.5)	
Housewife	00 (0.0)	70 (10.0)	71 (6.3)	
Student	225 (52.3)	328 (46.8)	552 (48.9)	
Self employed	93 (21.6)	151 (21.5)	244 (21.6)	
Employed	43 (10.0)	57 (8.1)	100 (8.9)	
Monthly income	*n* = 422	*n* = 670	*N* = 1,092	<0.0001
< 50,000FCFA (~100USD)	256 (60.7)	443 (66.1)	699 (64.0)	
50–100,000FCFA (~100-200USD)	92 (21.8)	170 (25.4)	262 (24.0)	
> 100,000FCFA (>200USD)	74 (17.5)	57 (8.5)	131(12.0)	

*BMI* body mass index, *SD* standard deviation, *FCFA* central African franc, *USD* United States Dollar, Apart from age and BMI (means compared using independent samples t-test), the rest of the characteristics are frequencies (percentage) compared using chi squared test

### Prevalence and age-trend in overweight and obesity

**Fig. 1:**
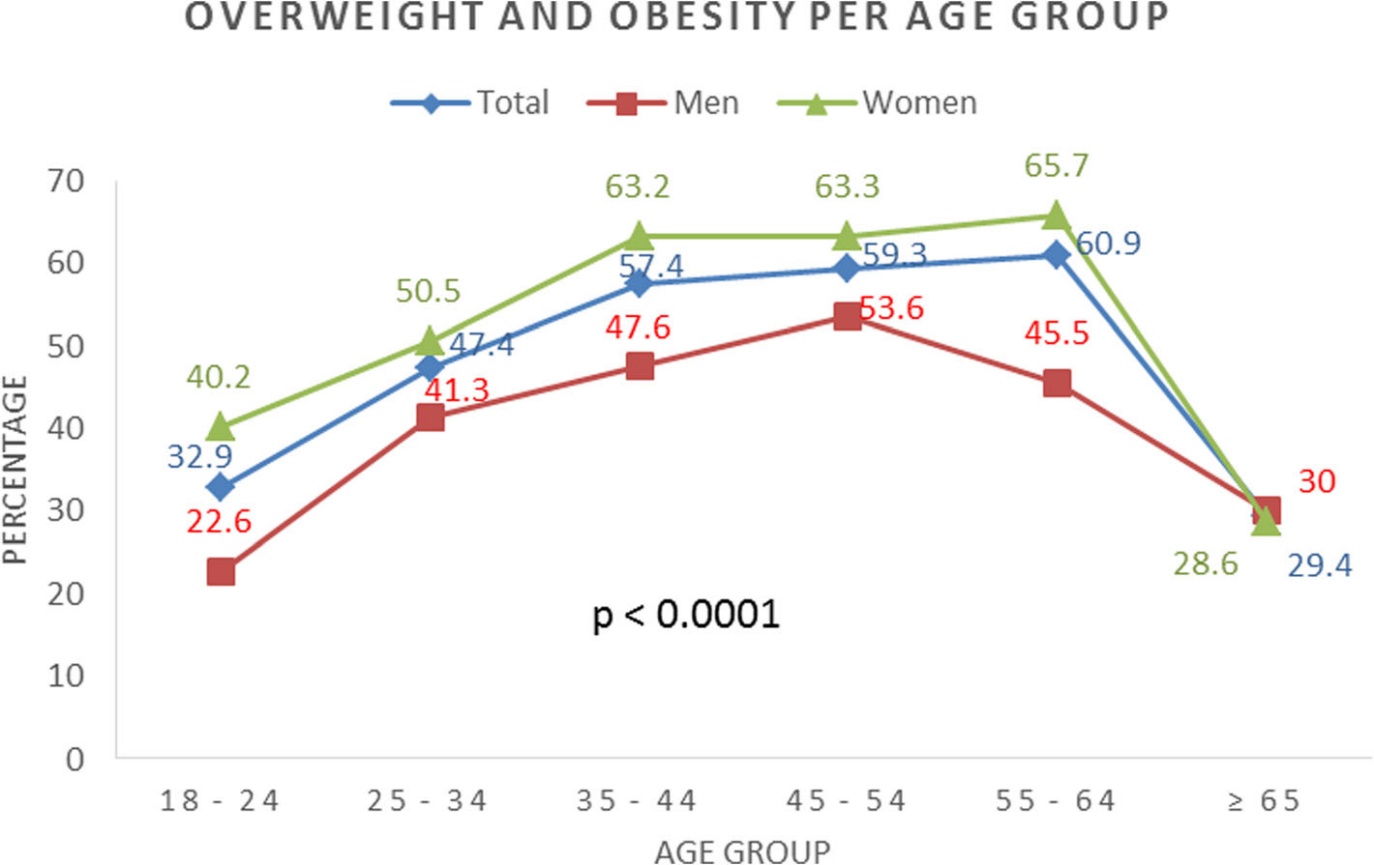
Trend in prevalence of overweight and obesity by sex and age group

The overall age-standardized prevalence of overweight and obesity was 36.5% (33.7–39.3) and 11.1% (9.3–12.9) while mean BMI was 25.3 ± 4.3 kg/m^2^. Women were heavier than the men with overweight and obesity rates of 37.4% and 13.2% vs. 30.6% and 5.1%; mean BMI of 25.8 kg/m^2^ vs. 24.4 kg/m^2^; both *p* < 0.0001. Overweight and obesity were more frequent with increasing monthly income (*p* < 0.0001). Similarly, there was higher prevalence in those with a family history of CVD (47.7% vs. 43.2%; *p* = 0.005). An increasing trend in prevalence of overweight and obesity existed across age groups with highest prevalence for men at age 45–54 years (53.6%) and women at age 55–64 years (65.7%), and declines thereafter (p-trend < 0.0001), see Table 2 and Fig. 1.

**Table 2:** Overall prevalence of overweight and obesity and according to demographic and clinical characteristics

Characteristics	Normal *n* = 628	Overweight *n* = 396	Obese *n* = 115	Total *N* = 1,139	*p*-value
Overall crude prevalence (95% CI)	55.1 (52.3–57.9)	34.8 (32.0–37.6)	10.1 (8.3–11.9)		
Age-adjusted prevalence (95% CI)	52.4 (49.5–55.3)	36.5 (33.7–39.3)	11.1 (9.3–12.9)		
BMI, mean ± SD, kg/m^2^	22.6 ± 1.6	27.0 ± 1.3	34.0 ± 6.4	25.3 ± 4.3	<0.0001
Gender	*n* = 628	*n* = 396	*n* = 115	*N* = 1,139	<0.0001
Male	280 (64.4)	133 (30.6)	22 (5.1)	435 (38.2)	
Female	348 (49.4)	263 (37.4)	93 (13.2)	704 (61.8)	
Educational level	624 (55.1)	394 (34.8)	114 (10.1)	*N* = 1,132	<0.0001
Up to secondary	124 (41.6)	125 (41.9)	49 (16.4)	298 (26.3)	
High school	119 (51.1)	97 (41.6)	17 (7.3)	233 (20.6)	
Diploma	56 (44.4)	47 (37.3)	23 (18.3)	126 (11.1)	
Undergraduate	280 (71.1)	97 (24.6)	17 (4.3)	394 (34.8)	
Post graduate	45 (55.6)	28 (34.6)	8 (9.9)	81 (7.2)	
Employment status	621 (55.0)	395 (35.0)	113 (10.0)	*N* = 1,129	<0.0001
Unemployed	71 (53.0)	48 (35.8)	15 (11.2)	134 (11.9)	
Retired	15 (53.6)	8 (28.6)	5 (17.9)	28 (2.5)	
Housewife	22 (31.0)	38 (53.5)	11 (15.5)	71 (6.3)	
Student	371 (67.2)	155 (28.1)	26 (4.7)	552 (48.9)	
Self employed	102 (41.8)	107 (43.9)	35 (14.3)	244 (21.6)	
Employed	40 (39.6)	39 (39.4)	21 (21.2)	100 (8.9)	
Monthly income	605 (55.5)	375 (34.4)	110 (10.1)	*N* = 1,090	<0.0001
< 50,000FCFA	418 (60.0)	222 (31.0)	57 (8.2)	697 (63.9)	
50-100,000FCFA	133 (50.8)	93 (35.5)	36 (13.7)	262 (24.0)	
> 100,000FCFA	54 (41.2)	60 (45.8)	17 (13.0)	131 (12.0)	
Perception of weight	620 (55.6)	383 (34.3)	112 (10.0)	*N* = 1,115	<0.0001
Underweight	32 (72.7)	10 (2.7)	2 (4.5)	44 (3.9)	
Normal	562 (58.7)	327 (34.1)	69 (7.2)	958 (85.9)	
Overweight	25 (23.4)	45 (42.1)	37 (34.6)	107 (9.6)	
Obese	0 (0.0)	1 (20.0)	4 (80.0)	5 (0.4)	
Physical activity frequency	585 (55.5)	361 (34.2)	109 (10.3)	*N* = 1,055	0.138
0-2times/week	274 (52.5)	188 (36.0)	60 (11.5)	522 (49.5)	
≥ 3times/week	311 (58.3)	173 (32.5)	49 (9.2)	533 (50.5)	
Healthy food	615 (54.9)	390 (34.8)	115 (10.3)	*N* = 1,120	0.183
Not every day	518 (54.2)	338 (35.4)	100 (10.5)	956 (85.3)	
Every day	97 (59.1)	52 (31.7)	15 (9.1)	164 (14.7)	
Family history of CVD	627 (55.3)	391 (34.5)	115 (10.2)	*N* = 1,133	0.005
Yes	193 (52.3)	123 (33.3)	53 (14.4)	369 (32.6)	
No	434 (56.8)	268 (35.1)	62 (8.1)	764 (67.4)	
Self-reported hypertension	626 (55.3)	391 (34.5)	115 (10.2)	*N* = 1,132	<0.0001
Yes	11 (1.8)	12 (3.1)	10 (8.7)	33 (2.9)	
No	615 (98.2)	379 (96.9)	105 (91.3)	1,099 (97.1)	
Self-reported diabetes	626 (55.3)	391 (34.5)	115 (10.2)	*N* = 1,132	0.015
Yes	4 (0.6)	3 (0.8)	4 (3.5)	11 (1.0)	
No	622 (99.4)	388 (99.2)	111 (96.5)	1,121 (99.0)	

*SD* standard deviation, *CI* confidence interval, *CVD* cardiovascular disease, group comparisons were done using chi squared test (for proportions) and independent samples t-test (for means)

### Factors associated with overweight and obesity

In the overall population, factors associated with overweight and obesity in multivariable analysis were; female gender, age >31 years, low level of education, being married and high monthly income (Table 3). In gender stratified analysis for women; low education [aOR: 1.91 (1.34–2.71), *p* <0.0001], age > 31 years [1.85 (1.23–2.55), *p* = 0.001], being married [2.30 (1.61–3.41), *p* <0.0001], high monthly income [2.25 (1.19–4.24), 0.012], physical inactivity [1.62 (1.20–2.21)), *p* = 0.005] were independently associated with overweight and obesity while older age [2.43 (1.50–3.73), *p* <0.0001] and being married [1.85 (1.13–2.89), *p* = 0.011] were associated with obesity alone. In men; older age [1.83 (1.11–3.02), *p* = 0.018] and being married [3.42 (1.90–6.13), *p* < 0.0001] for overweight and obesity, and being married [3.07 (1.20–8.71), *p* = 0.032] for obesity alone, were the independent associations in multivariable analysis (Tables 4 and 5).

**Table 3:** Factors associated with overweight and obesity in the overall study population

Characteristics	Overweight and obesity (*N* = 511)				Obesity alone (*N* = 115)			
	Bivariate analysis		Multivariable analysis (*R* ^*2*^ = 0.12)		Bivariate analysis		Multivariable analysis (*R* ^*2*^ = 0.10)	
	O.R. (95% CI)	*p*-value	aO.R. (95% CI)	*p*-value	O.R. (95% CI)	*p*-value	aO.R. (95% CI)	*p*-value
Sex								
Male	Ref		Ref		Ref		Ref	
Female	1.85 (1.44–2.36)	<0.0001	2.01 (1.58–2.76)	<0.0001	2.87 (1.78–4.65)	<0.0001	3.20 (1.93–5.59)	<0.0001
Age categories								
≤ 31 years	Ref		Ref		Ref		Ref	
> 31 years	2.34 (1.84–2.99)	<0.0001	1.97 (1.41–2.48)	0.001	3.81 (2.45–5.93)	<0.0001	3.21 (1.86–5.28)	<0.0001
Level of education								
High (diploma + university)	Ref		Ref		Ref		Ref	
Low-moderate (secondary + high school)	2.10 (1.62–2.60)	<0.0001	1.52 (1.14–2.03)	0.005	1.64 (1.11–2.43)	0.013	0.91 (0.58–1.49)	0.571
Married								
No	Ref				Ref		Ref	
Yes	3.65 (2.75–4.85)	<0.0001	3.11 (2.21–4.01)	<0.0001	2.97 (2.00–4.40)	<0.0001	2.10 (1.60–3.51)	<0.0001
Monthly income								
< 50,000 F (< USD100)	Ref		Ref		Ref		Ref	
50–100,000 F (USD100–200)	1.45 (1.09–1.93)	0.010	1.32 (1.01–1.69)	0.331	1.68 (1.10–2.89)	0.042	1.38 (0.87–2.17)	0.235
> 100,000 F (> USD 200)	2.14 (1.46–3.12)	<0.0001	2.11 (1.46–3.61)	<0.0001	1.79 (1.15–2.81)	0.010	1.45 (0.76–2.77)	0.262
Frequency of physical activity (min. of 30mins)								
≥ 3 times/ week	Ref		Ref		Ref		Ref	
0–2 times/ week	1.27 (0.99–1.62)	0.056	1.27 (0.94–1.61)	0.120	1.31 (0.91–1.90)	0.228	1.21 (0.74–1.89)	0.367
Alcohol Use								
No	Ref				Ref			
Yes	1.17 (0.98–1.92)	0.602	-		0.83 (0.46–1.48)	0.520	-	
Smoking status								
No	Ref				Ref			
Former	0.65 (0.29–1.12)	0.823	-		0.42 (0.16–3.01)	0.501	-	
Current	0.87 (0.31–2.01)	0.694	-		0.68 (0.23–2.35)	0.328	-

**Table 4:** Factors associated with overweight and obesity in adult men and women in Buea, Southwest region, Cameroon s2016

Characteristics	Women (*n* = 700)				Men (*n* = 431)			
	Bivariate analysis		Multivariable analysis (*R* ^*2*^ = 0.16)		Bivariate analysis		Multivariable analysis (*R* ^*2*^ = 0.12)	
	O.R. (95% CI)	*p*-value	aO.R. (95% CI)	*p*-value	O.R. (95% CI)	*p*-value	aO.R. (95% CI)	*p*-value
Age categories								
≤ 31 years	Ref		Ref		Ref		Ref	
> 31 years	2.46 (1.82–3.34)	<0.0001	1.85 (1.23–2.55)	0.001	2.29 (1.54–3.43)	<0.0001	1.83 (1.11–3.02)	0.018
Level of education								
High (diploma + university)	Ref		Ref		Ref		Ref	
Low-moderate (secondary + high school)	2.44 (1.80–3.30)	<0.0001	1.91 (1.34–2.71)	<0.0001	1.44 (0.97–2.15)	0.071	1.11 (0.66–1.76)	0.764
Married								
No	Ref		Ref		Ref		Ref	
Yes	3.24 (2.30–4.58)	<0.0001	2.30 (1.61–3.41)	<0.0001	3.88 (2.32–6.49)	<0.0001	3.42 (1.90–6.13)	<0.0001
Monthly income								
< 50,000 F (< USD100)	Ref		Ref		Ref		Ref	
50–100,000 F (USD100–200)	1.68 (1.17–2.40)	0.005	1.51 (1.01–2.19)	0.034	1.10 (0.65–1.82)	0.736	1.01 (0.59–1.69)	0.991
> 100,000 F (> USD 200)	2.23 (1.25–3.96)	0.006	2.25 (1.19–4.24)	0.012	2.65 (1.56–4.51)	0.001	1.94 (1.01–2.83)	0.202
Frequency of physical activity (min. of 30mins)								
≥ 3 times/ week	Ref		Ref		Ref			
0–2 times/ week	1.38 (1.01–1.87)	0.041	1.62 (1.20–2.21)	0.005	0.89 (0.58–1.35)	0.575	-	
Alcohol Use								
No	Ref				Ref		Ref	
Yes	1.10 (0.69–1.92)	0.570	-		2.17 (1.36–3.45)	0.001	1.73 (1.05–2.83)	0.301
Smoking status								
No	Ref				Ref			
Former	0.84 (0.28–2.52)	0.756	-		0.56 (0.40–1.16)	0.527	-	
Current	0.98 (0.19–4.89)	0.980	-		0.37 (0.01–1.21)	0.326	-

**Table 5:** Variables associated with obesity alone in adult men and women from Buea, Southwest region of Cameroon, 2016

Characteristics	Women (*n* = 700)				Men (*n* = 431)			
	Bivariate analysis		Multivariable analysis (*R* ^*2*^ = 0.14)		Bivariate analysis		Multivariable analysis (*R* ^*2*^ = 0.11)	
	O.R. (95% CI)	*p*-value	aO.R. (95% CI)	*p*-value	O.R. (95% CI)	*p*-value	aO.R. (95% CI)	*p*-value
Age categories								
≤ 31 years	Ref		Ref		Ref		Ref	
> 31 years	4.11 (2.46–6.85)	<0.0001	2.43 (1.50–3.73)	<0.0001	2.72 (1.11–6.80)	0.033	1.51 (0.51–4.55)	0.465
Level of education								
High (diploma + university)	Ref		Ref		Ref			
Low-moderate (secondary + high school)	1.82 (1.16–2.85)	0.009	1.31 (1.08–2.61)	0.121	0.91 (0.38–2.18)	0.840	-	
Married								
No	Ref		Ref		Ref		Ref	
Yes	2.35 (1.51–3.67)	<0.0001	1.85 (1.13–2.89)	0.011	4.33 (1.79–10.43)	0.001	3.07 (1.20–8.71)	0.032
Monthly income								
< 50,000 FCFA (< USD100)	Ref		Ref		Ref			
50–100,000 FCFA (USD100–200)	2.00 (1.23–3.27)	0.006	1.13 (1.02–2.98)	0.103	0.51 (0.18–1.43)	0.199	-	
> 100,000 FCFA (> USD 200)	2.06 (0.99–4.26)	0.050	1.20 (1.10–3.15)	0.245	0.52 (0.14–1.89)	0.319	-	
Frequency of physical activity (min. of 30mins)								
≥ 3 times/ week	Ref				Ref		Ref	
0–2 times/ week	1.01 (0.65–1.58)	0.950	-		1.97 (0.77–5.01)	0.155	1.28 (0.56–4.32)	0.352
Alcohol Use								
No	Ref				Ref			
Yes	1.02 (0.486–2.13)	0.936	-		1.07 (0.38–2.98)	0.896	-	
Smoking status								
No	Ref				Ref			
Former	0.55 (0.07–4.27)	0.566	-		1.42 (0.40–5.02)	0.590	-	
Current	0.87 (0.21–3.65)	0.803	-		0.65 (0.08–5.10)	0.682	-	

### Population attributable fraction estimates

Our estimates for PAF showed that overweight and obesity among women in the population could be attributed to marriage (21.8%), low level of education (11.8%), physical inactivity (8.7%) and high monthly income (6.4%), while 7.8%, 6% and 10.8% of the obesity among women could be attributed to marriage, having a family history of CVD and older age respectively. In men, 28.3% of overweight and obesity could be attributed to marriage, which accounted for about 7% of obesity alone (Table 6).

**Table 6:** Population attributable fraction estimates for obesity by gender in Buea, Southwest region, Cameroon

Factor	*P*(95%CI)	Adjusted RR (95% CI)	PAF (95% CI)
Overweight and Obesity			
Women			
Age > 31 years	61.7 (40.0–83.4)	1.24 (1.05–1.42)	11.9 (1.9–24.7)
Marriage	70.3 (41.9–98.6)	1.45 (1.26 – 1.63)	21.8 (8.6–38.1)
Low education	61.9 (57.2–66.6)	1.23 (1.03–1.43)	11.6 (1.7–20.0)
High income	55.9 (43.5–68.3)	1.13 (1.01–1.25)	6.4 (0.4–13.6)
Physical inactivity	54.2 (32.6–75.8)	1.19 (1.02–1.36)	8.7 (0.6–20.1)
Men			
Age > 31 years	46.0 (19.5–72.5)	1.25 (1.02–1.48)	9.2 (0.4–23.5)
Marriage	62.3 (32.1–92.5)	1.83 (1.49–2.16)	28.3 (10.6–49.7)
Obesity alone			
Women			
Age > 31 years	20.9 (15.5–26.5)	2.06 (1.55–2.55)	10.8 (5.4–16.1)
Marriage	20.8 (7.9–18.1)	1.60 (1.12–2.08)	7.8 (0.8–9.4)
Men			
Marriage	12.9 (4.4–21.6)	2.03 (1.12–2.68)	6.7 (1.2–13.5)

*P* prevalence of obese cases exposed to given risk factor, *RR* relative risk, *PAF* population attributable fraction

## Discussion

Determinants for overweight and obesity have previously been explored in the Cameroonian population; however, how much overweight and obesity in the general population can be attributed to these determinants has not been studied in Cameroon. In this population-based study, we found that over a third of participants were overweight or obese. The overweight and obesity burden in this population was partly attributable to low levels of education, older age, high monthly income, physical inactivity and being married.

These findings suggest an increasing trend in overweight and obesity in Cameroon, as previous reports in Cameroon showed just over a quarter of the population was overweight [34, 35]. Our findings are similar to reports of a recent systematic review from Nigeria which found rates of 20–35% and 8.1–22% for overweight and obesity respectively [36]. Higher rates of obesity have been reported in South Africa, with rates twice as high as our findings [9]. As expected, women were found to be significantly heavier than men, which confirm previous local reports [18, 30, 32] and findings elsewhere [10, 31, 33]. With respect to age, we noticed an overall increasing trend in prevalence of overweight and obesity that peaked at age group 45–54 years and 55–64 years for men and women respectively. Similar trends were also observed elsewhere in Cameroon [35, 37], and in Uganda [38]. Most of these studies were from urban settings, with a few involving rural participants, while our study was composed mainly of semi-urban dwellers, who tend to have a higher prevalence of obesity. Furthermore, an overall tendency to overweight was observed in our study population (mean BMI of 25.3 kg/m^2^). This was marginally higher than previously reported in Cameroon [34, 35]. While the variance may not be striking, it should be noted that the majority of our study population was made up of young adults, almost a decade younger than previous reports in Cameroon [34]. This suggests an expansion (including younger people) in the burden of overweight and obesity and consequently an increased risk of NCDs in the younger population. This could translate into a high disease burden in the future, if nothing is done to curtail the rise in overweight and obesity rates.

We found that older age, being married, a low level of education, high monthly income and physical inactivity were potential determinants of overweight and obesity among the women, while being married was the main determinant in men. Physical inactivity explained about a tenth of the overweight and obesity among women in this population. This could be due to the adoption of western lifestyles and sedentary behaviours seen to accompany the epidemiologic transition most SSA countries are currently facing. Previous subnational studies in Cameroon [13, 18] have shown an association between physical inactivity and overall obesity prevalence, though less clearly in women, which contrasts our findings. A recent study from South Africa found that lack of exercise accounted for 15% of the obesity [9]. Larger nationally representative studies in Cameroon exploring sex specificities in relation to physical activity levels, obesity and overall metabolic health have a potential to better inform understanding.

With respect to education, low levels of education accounted for 12% of the overweight and obesity among women. Studies from other African countries have found similar associations between low level of education and obesity [9, 39]. This could in part be explained by the fact that more literate individuals are likely to be more informed and potentially tend to adopt healthier lifestyles [40]. Interestingly, we found in our study that though women with low education were more affected, a high monthly income explained more of the overweight and obesity. This is in line with prior studies in Cameroon [13] and Tanzania [41] which found a positive relationship between adiposity and socio-economic status. Our findings are analogous to those of Roskam et al. who found that individuals with low levels of education were more likely to have increased waist lines in Europe [42]. Thus, our results suggest a particular situation with patterning of overweight and obesity as regards educational levels of Cameroonians, with individuals having low levels of education being disadvantaged. Larger nationwide studies are however welcome to explore from a broader perspective, the relationship of obesity and education in the Cameroonian population.

Studies elsewhere have suggested that increasing socio-economic development is associated with increased affordability of cheap energy-dense foods with the major consequence of these being among those in the lower socio-economic groups [43]. Cameroon has steadily experienced increasing per capita dietary intake from 2,001 Kcal/Day in 1962–2,269 Kcal/Day in 2007 and 2,586 Kcal/Day in 2011. Similarly, per capita consumption of sugar and starchy foods was 104 and 419Kcal/day respectively in 2011 [24]. This recent surge and increasing trend in dietary energy intake with relatively low activity levels paralleling increase in other related risk factors like high blood pressure [44], support the unhealthy influence (adoption of ‘western’ diets and physical activity patterns) of urbanization in Cameroon and other low income countries. However, contrary to western countries where women in the higher socio-economic groups have the tendency to maintain small sizes [45], women with highest monthly income in our study had over twice the odds of being overweight or obese compared to those with low incomes.

While there is evidence about the potential health benefits associated with marriage [46], our findings suggest that marriage is also associated with overweight and obesity. Being married explained about a quarter of overweight and obesity and 15% of obesity alone, in both men and women. This was comparable to findings from South Africa, where being married was a significant determinant for obesity, especially among males [9], and to findings from a study among Greek adults [47]. Whilst there have been suggestions explaining this association in western countries [47], there is need for more in depth exploration of this relationship in the Cameroonian or African context.

Factors such as ageing seem particular in terms of preventive interventions as it is a non-modifiable risk factor, essentially reflecting the potential cumulative exposure to other behavioural and environmental factors. However, adequate counselling of young couples (and even prenuptial) with genetic risk (family histories) to adopt healthy lifestyles may assist in reducing the obesity burden in subsequent generations.

Our study has some limitations. The cross sectional design used precluded the actual exploration of causality for obesity in the population but rather obtained associations using odds ratios, which inherently seem inappropriate for PAF estimations. We therefore used rigorous statistical methods to obtain relative risks that were adjusted for potential confounders, and used these to estimate PAFs and the uncertainty around the estimates. In addition, we used a formula for PAF calculations which accounts for confounding. It is possible that with some household visits for recruitment occurring during the day, we may have missed some adults who were at their places of work. However, we attempted to avert this by alternating between day and evening visits to maximise participation in the study. Our study provides recent updates on the burden of obesity in semi-urban Cameroon and is among the few to have explored population attributable fractions for determinants of obesity in sub-Saharan Africa, and the first of its kind in Cameroon.

## Conclusions

Our study revealed a high prevalence of overweight and obesity affecting a third of the population and has provided pioneer evidence on its determinants. Population-based interventions promoting physical activity and health education targeting people with relatively low education, high income earners and couples are potential avenues to control the rapidly growing overweight and obesity epidemic in low-income countries.

## Abbreviations

BMI: body mass index
CVD: cardiovascular disease
PAF: population attributable fraction
RR: relative risk
WHO: World health organization
